# A Neural Network Based Approach to Inverse Kinematics Problem for General Six-Axis Robots

**DOI:** 10.3390/s22228909

**Published:** 2022-11-18

**Authors:** Jiaoyang Lu, Ting Zou, Xianta Jiang

**Affiliations:** 1 Department of Mechanical Engineering, Memorial University of Newfoundland, St. John’s, NL A1B 3X5, Canada; 2 Department of Computer Science, Memorial University of Newfoundland, St. John’s, NL A1B 3X5, Canada

**Keywords:** inverse kinematics, general six-axis robot, neural network, numerical error minimization

## Abstract

Inverse kinematics problems (IKP) are ubiquitous in robotics for improved robot control in widespread applications. However, the high non-linearity, complexity, and equation coupling of a general six-axis robotic manipulator pose substantial challenges in solving the IKP precisely and efficiently. To address this issue, we propose a novel approach based on neural network (NN) with numerical error minimization in this paper. Within our framework, the complexity of IKP is first simplified by a strategy called joint space segmentation, with respective training data generated by forward kinematics. Afterwards, a set of multilayer perception networks (MLP) are established to learn from the foregoing data in order to fit the goal function piecewise. To reduce the computational cost of the inference process, a set of classification models is trained to determine the appropriate forgoing MLPs for predictions given a specific input. After the initial solution is sought, being improved with a prediction error minimized, the refined solution is finally achieved. The proposed methodology is validated via simulations on Xarm6—a general 6 degrees of freedom manipulator. Results further verify the feasibility of NN for IKP in general cases, even with a high-precision requirement. The proposed algorithm has showcased enhanced efficiency and accuracy compared to NN-based approaches reported in the literature.

## 1. Introduction

By virtue of recent breakthroughs in data communications, artificial intelligence and hardware, robots have experienced significant improvement in intelligent levels that prompt wide applications in daily lives, especially for repetitive, time-consuming, and high-risk tasks [1,2,3]. In such situations, the task is prescribed by a sequence of poses in the Cartesian space whereas the robot is controlled in the joint space. Hence, a problem termed the Inverse Kinematics Problem in robotics arose [4].

The IKP, together with its counterpart, the forward kinematic problem (FKP), gives a complete mapping between joint variables and the pose of the end-effector. Playing a key role in the robot trajectory planning and motion control, a solution for IKP with high precision is desirable. While FKP can be solved by the well known Denavit–Hartenberg (D-H) conventions with ease, handling IKP is much more challenging. Unlike FKP, different combinations of joint angles may lead to a same pose of the end-effector, resulting in multiple potential solutions. In fact, it has been proven that up to 16 inverse kinematics solutions can be expected for a general six degrees of freedom (DoFs) robot [5]. Furthermore, in order to reach any pose in the real space theoretically, robots are usually designed with at least six joints. Kinematic equations under such high DoFs are always highly coupled and non-linear, which poses substantial challenges for achieving the solution.

Up to now, only robots with some special geometric structures, such as spherical wrist, are reported to have closed-form solutions [6,7]. The IKP of this type can be decoupled and these robots are, hence, named decoupled robots. For others, e.g., the general type robot, the closed-form solution does not exist. Numerous approaches based on different principles, which can be categorized as geometric [8,9], algebraic [10,11], and numerical methods [12,13,14], have been proposed. Unfortunately, all these methods suffer from inherent bottlenecks. Geometric methods are robot-dependent and only feasible for robots with simple structures. On the other side, algebraic methods fail to give an explicit expression of the final solution and the whole elimination processes are extremely tedious, making them hard and even impractical to be implemented under high-DoF circumstance. For numerical methods, apart from some possible drawbacks, such as singularity, joint limitations, and local optimum problem, the sought solution is deeply influenced by the initial guess set at the beginning. Strategies for choosing a suitable starting point add extra work.

Due to the limitations of the above traditional approaches, soft-computing methods have gained more attentions and become increasingly popular in recent years. Via non-linear function approximators or heuristic roots searching process [15], these algorithms can explore the answer without a priori knowledge of the robot models, among which neural network (NN) is a choice in fashion for IKP. Work of Karlik and Serkan on a 6 DoF robot was a typical early study [16]. Two architectures of artificial neural network (ANN)—one hidden layer with six outputs and two hidden layers with single output—were compared and the total errors were below 2% and 0.6%, respectively. However, the solutions were only represented within the joint space and not transformed into the Cartesian space, making the results less meaningful. Later, in [17], the performance of the radial basis function (RBF) network and the back propagation (BP) network for the IKP was compared. As the RBF network was concluded to outperform the BP network with a maximum error of 0.08°, only three target poses were used during the test. Thus, room for improvement accompanies the result due to relatively low test data. To increase the reliability of the NN predictions, in [18], three recurrent networks were trained in parallel to form the prediction system. While the mean squared error (MSE) of the whole system was 0.015687, the reliability was claimed to be 99.999993664% after training. Although this reliability seems perfect, it is computed by simple multiplications of the reliabilities from each trained network, ignoring the fact that the probability behind each component is not independent. Chiddarwar and Babu [19] trained a novel RBF network to tackle IKP. Unlike traditional networks using the goal pose as input and joint state as output, their model was additionally fed with incremental change of the pose in order to predict the increment of joint angles. Simulation results on KUKA-Kr robot revealed the improvement of their model over traditional networks. To get rid of the kinematic singularities, a single hidden layer NN was trained to solve the IKP and the kinematics Jacobian simultaneously in [20]. Implementing on a 6-DOF manipulator, maximum IK errors of 4.81% in the joint space, as well as 6.72% and 5.79% for position and orientation, respectively, in the Cartesian space, were observed. Köker investigated the idea of combining NN and genetic algorithm (GA) for IKP [21]. The integer portion of the solution was first obtained from NNs while the floating-point portion was further improved by GA. With this workflow, the precision of the solver can reach the micrometer level. Take a further step, Köker [22] optimized the previous GA error minimization procedure by introducing simulated annealing algorithm as a genetic operator. Hence, the number of epochs reaching the optimal solution was reduced to a half of the method without using annealing algorithm. Feng et al. proposed a novel approach to solve IKP with the single hidden layer feedforward neural networks (SLFNs) in [23]. Within their scheme, an electromagnetism-like method was used to generate the training within the joint subspace while the extreme learning machine analytically determined the weights of the SLFNs instead of back propagation (BP) training. Although this approach was proved to be highly precise, the SLFNs need to be retrained every time given a new target pose, which limited its application. Almusawi et al. [24] took the current joint state into consideration during implementing ANN and verified their modification can be helpful to achieve better performance for ANN system. In [25], the performance of NN for IKP was compared with those of GA and adaptive neuro-fuzzy inference system (ANFIS). The results from the drawing spring shape task demonstrated that NN was much more faster than GA and had a better accuracy in contrast to ANFIS. A model based on generative adversarial networks (GAN) was proposed for IKP and inverse dynamics (ID) in [26]. A comparative test was conducted between the proposed model and the traditional feedforward network (FFN). Ignoring the ID part, GAN showed no obvious advantages over FFN for IKP and the minimum MSE was 0.0256 in their work. Toquica et al. [27] utilized MLP to determinate the IKP solution of a 6-DoF industrial robot, as well as the best architecture for the NN; they also discussed the effect of choosing different solvers, activation functions, and hidden layers sizes. However, error minimization was not considered for their NN prediction and the mean absolute error for the last joint was up to 1.56557°. In [28], a 3-DoF robot arm was modeled with unit quaternions instead of D-H convention for IKP training. After learning the respective features with an MLP, the maximum IKP prediction error is 0.0669° in the joint space. Additionally, some researchers lay their emphases on the training process, and the heuristic algorithms are applied to train the model for IKP, such as the PSO-optimized NN in [29] and the FOA-optimized NN in [30]. Maximum observed prediction error for these two models were −0.1859° and 0.1271°, respectively.

As a brief summary of the previous work, we sort and summarize their features in Table 1. It can be observed that, despite the high precision showcased by their IKP solutions, a common limitation accompanies: the robot models being studied are simple, being either decoupled or low DoF. Testing the learning-based algorithms on these robot models is inappropriate. On the one hand, for these decoupled or low-DoF models, the compact, elegant and high-efficiency analytical IKP solutions are always the optimal choice, making the learning-based approaches somewhat meaningless in this scenario. Furthermore, inverse kinematics functions under low-DoF or decoupled case are relatively simple and easier to be learnt from training data. Hence, performance of these NN-based methods on general high-DoF robots, the IKP of which deserves most attentions, is still not clear and worth discussing.

In this paper, a novel NN-based approach combining the regression and classification model was proposed to solve the IKP of general six-axis robots. Due to the complexity of the problem, the underlying IK function was first simplified by joint space segmentation, with the respective training data generated via FKP on each subspace. Then, the prediction system, comprising several sets of NNs, was trained on these data to learn the problem in piece. Meanwhile, a set of classification models was trained to determine which foregoing NNs, representing different joint spaces, should be selected for prediction given a desired pose. Considering the precision requirement of some sensitive tasks, an iterative process was applied to minimize the prediction error subsequently. With this configuration, we hypothesized that our algorithm can achieve high precision and stability. This hypothesis is verified by simulations with comparison to other models reported in the literature.

Our contributions are twofold: On the one hand, the performance of neural network methods for IKP is further explored and improved for six-joint general robots. Due to the complexity of our robot model, the existing methods in the literature, which fit the problem directly, are not capable of solving the IKP of the aforementioned type of robots accurately. Our novel NN-based method as a robust integration of the regression and classification model can solve the problem efficiently and the precision of the algorithm is secured by the idea of joint space segmentation. In addition, an error minimization method is investigated based on the classic Newton–Raphson (NR) algorithm. One novelty of this approach is its robustness in minimizing the singularity in the IKP problem. Moreover, the method also has advantages in the sense of overcoming underlying drawbacks associated with the commonly used error minimization algorithm, such as the efficiency.

The rest of this paper is organized as follows: Section 2 covers the robot model and kinematic analysis. Section 3 gives a brief introduction about the mathematical model of neural networks and the Newton–Raphson based error minimization method. In Section 4, some details during implementation are covered while the simulation results are shown and discussed in Section 5. Finally, Section 6 concludes the paper.

## 2. Robot Architecture and Kinematic Modeling

Robot kinematics specifies the relationship between joint angles and the pose of the end-effector, which contains two parts: forward kinematics and inverse kinematics. As mentioned before, the complexity of kinematics, especially the IKP, is closely associated with robot architecture. In our work, to study the IKP of a general six-joint manipulator, Xarm6 is chosen, a six-joint manipulator with an offset wrist from Ufactory. With this architecture, analytical solution of the IKP cannot be expected and, thus, the presented algorithms are universal for any 6-DoF robot.

**Figure 1:**
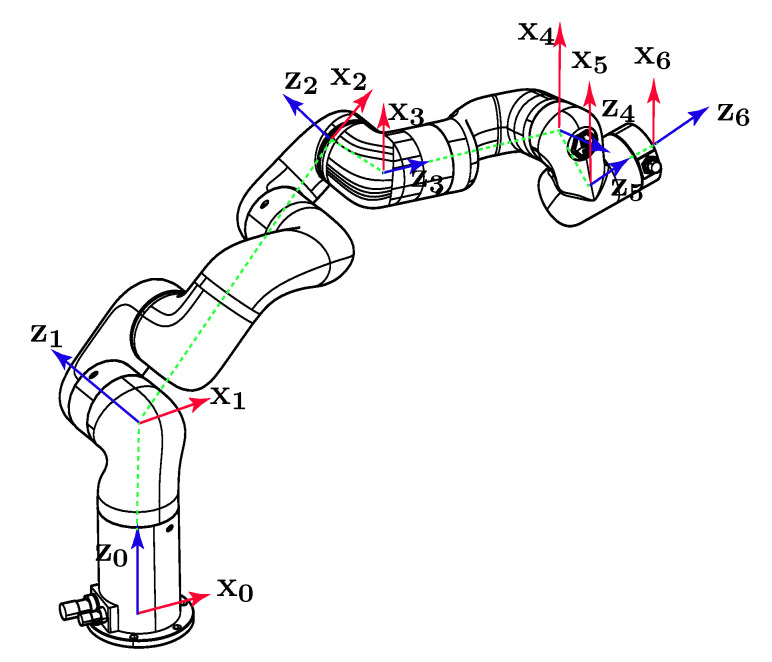
Frame assignment of Xarm6.

In forward kinematics, the pose of the end-effector can be calculated with D-H conventions and homogeneous matrix once the joint variables are obtained. Figure 1 illustrates the assignment of D-H frames on Xarm6, while Table 2 lists related D-H parameters in the attached frames, where *a*, α, *d*, θ denote link length, link twist, link offset, and joint variable, respectively.

With D-H parameters, the transformation matrix $A_{i}$ from frame i−1 to frame *i* is defined as

(1) $$A_{i}=\left[\begin{array}{ccccccc} c\theta_{i} & \; & -s\theta_{i}c\alpha_{i} & \; & s\theta_{i}s\alpha_{i} & \; & a_{i}c\theta_{i}\\ s\theta_{i} & \; & c\theta_{i}c\alpha_{i} & \; & -c\theta_{i}s\alpha_{i} & \; & a_{i}s\theta_{i}\\ 0 & \; & s\alpha_{i} & \; & c\alpha_{i} & \; & d_{i}\\ 0 & \; & 0 & \; & 0 & \; & 1 \end{array}\right]$$

where c(·) and s(·) stand for cos(·) and sin(·), respectively.

Then, the pose of the end-effector, represented by homogeneous matrix $T_{6}$, can be calculated as:

(2) $$T_{6}=A_{1}A_{2}A_{3}A_{4}A_{5}A_{6}=\left[\begin{array}{ccc} R_{6} & \; & d_{6}\\ 0 & \; & 1 \end{array}\right]$$

in which $R_{6}$ and $d_{6}$ are the 3 × 3 rotation matrix and the 3 × 1 position vector of the end-effector with respect to the base frame.

Since the nine entries in $R_{6}$ are not independent, it is advantageous to obtain the minimum representation of the pose rather than using the homogeneous matrix directly. Obtaining the position of the end-effector is quite straightforward, as this can be read directly from $d_{6}$. The expression of orientation, nevertheless, is more complex and Euler angles, roll pitch yaw, and quaternions are the three most commonly used notations. In our work, the orientation of a pose is described by Euler angles ϕ, θ, ψ with ZYZ sequence. Hence, the pose of the end-effector is represented with the following form:

(3) $$p={\left[x,y,z,\phi ,\theta ,\psi \right]}^{T}$$

in which *x*, *y*, and *z* denote the position of the end-effector in the base frame, ϕ, θ, and ψ being the Euler angles.

For practical revolute joints, unlike the ideal model which can rotate within 0–360°, the physical restrictions limit their motions. For our robot, the joint limitations are:

(4) $$\begin{array}{cc} \; & 0\leq \theta_{1}\leq 360{}^{\circ},\\ \; & 0\leq \theta_{2}\leq 90{}^{\circ},\\ \; & -180\leq \theta_{3}\leq -90{}^{\circ},\\ \; & 0\leq \theta_{4}\leq 180{}^{\circ},\\ \; & 0\leq \theta_{5}\leq 180{}^{\circ},\\ \; & 0\leq \theta_{6}\leq 360{}^{\circ}, \end{array}$$

## 3. Neural Network with Error Minimization

### 3.1. Neural Network

The neural network is an algorithm mimicking the operations of a human’s brain to explore the underlying pattern among sets of data. It has been proven that, with a suitable amount of neurons, a two hidden layers network has the ability to approximate any non-linear function with an arbitrary error, regardless of the order of the problem theoretically [31]. For our IKP problem, MLP is selected by virtue of computational robustness and low computational cost. A brief introduction to the mathematical model of MLP is given as below.

**Figure 2:**
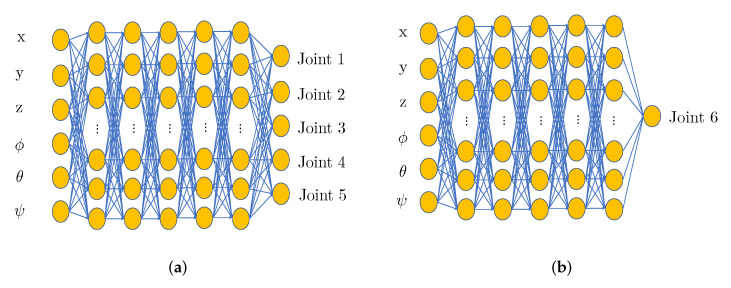
NN architecture for IKP. (**a**) NN for the first five joints; (**b**) NN for the last joint.

A MLP is composed of an input layer, an output layer, and several hidden layers. Figure 2 demonstrates two examples of MLP with five hidden layers. Each layer contains certain amounts of basic computational units called neurons. For each neuron, there exists an activation function g(·), the relationship between the input and output of the neuron being represented as:

(5) a=g(z)

where *a* and *z* are the output and input of the neuron, respectively.

Then, the relationship between the outputs of two adjacent layers can be modeled as:

(6) $$a^{i+1}=g\left(\Theta^{i}a^{i}\right)$$

where $a^{i}$ is the output of layer *i*, $\Theta^{i}$ being the weight matrix between layers *i* and i+1. By using Equation (6), the prediction or hypothesis of MLP can be calculated with a chain process.

To seek the optimum set of weights fitting the proposed inverse kinematics function, the cost function is defined as:

(7) $$J\left(\Theta \right)=\frac{1}{m}\sum_{i=1}^{m}\sum_{k=1}^{n}{\left[Y_{k}^{i}\left(p_{i}\right)-y_{k}^{i}\left(p_{i}\right)\right]}^{2}$$

where *m* is the amount of the training data, *n* is the number of the outputs, $Y_{k}^{i}\left(p_{i}\right)$ is the *k*th output in the *i*th set of training data, $y_{k}^{i}\left(p_{i}\right)$ is the *k*th prediction of the network for the *i*th set of input. The training method applied in our study is Levenberg–Marquardt (L-M) and Adam for the prediction and classification system, respectively, with more details found in [32,33,34].

### 3.2. Numerical Error Minimization

Despite NN’s robustness in fitting any non-linear function without boundary on the minimum error ideally, some limitations pose substantial challenges in achieving the optimal performance, including the problem complexity, limited training time, scarce training data, and restricted computational resources. From previous works, for IKP, it is challenging to make a prediction with the precision of micrometer level on a 6-DOF decoupled manipulators [21,25,27,35], let alone our more complex system. Thus, apart from establishing improved NN architectures, finding a suitable method to refine the primary solution from trained neural networks would be an ideal choice. In order to avoid increasing the overall computational time, we choose a straightforward, efficient root seeking approach, the Newton–Raphson method, whose error minimization process is shown below.

With the minimum representation of the end-effector pose defined in Equation (3), we can write the FK equation as

(8) $$p={\left[x,y,z,\phi ,\theta ,\psi \right]}^{T}=f\left(q\right)$$

where f(·) is the forward kinematic function, while q is the vector of joint variables.

Then, denoting the goal pose as $p_{g}$, we construct a new function g(q) as

(9) $$g\left(q\right)=f\left(q\right)-p_{g}$$

Let $q_{0}$ be the initial solution of the NN. Expanding g(q) with Taylor series at $q_{0}$ yields:

(10) $$g\left(q\right)=f\left(q_{0}\right)-p_{g}+J_{a}\left(q_{0}\right)\left(q-q_{0}\right)+h.o.t.$$

where $J_{a}$ is the analytical Jacobian matrix.

Note that seeking the root of equation $p_{g}=f\left(q\right)$ is equivalent to finding the root of g(q)=0. Substituting g(q)=0 into Equation (10) and ignoring the higher order terms leads to:

(11) $$q\approx q_{0}+J_{a}^{-1}\left(q_{0}\right)\left[p_{g}-f\left(q_{0}\right)\right]$$

Hence, we can use the following iterative equation to minimize the error of the initial solution:

(12) $$q_{k+1}=q_{k}+\alpha J_{a}^{-1}\left(q_{k}\right)\left[p_{g}-f\left(q_{k}\right)\right]$$

where α is the step size. After each iteration, $q_{k}$ makes a step towards the expected solution and the process will stop after the error $|f\left(q_{k}\right)-p_{g}|$ meets the condition of convergence. In our analysis, the convergence error is set 0.001 mm for position, and 0.001 rad for orientation, which are high-level criteria for practical tasks [21].

## 4. Implementation

**Figure 3:**
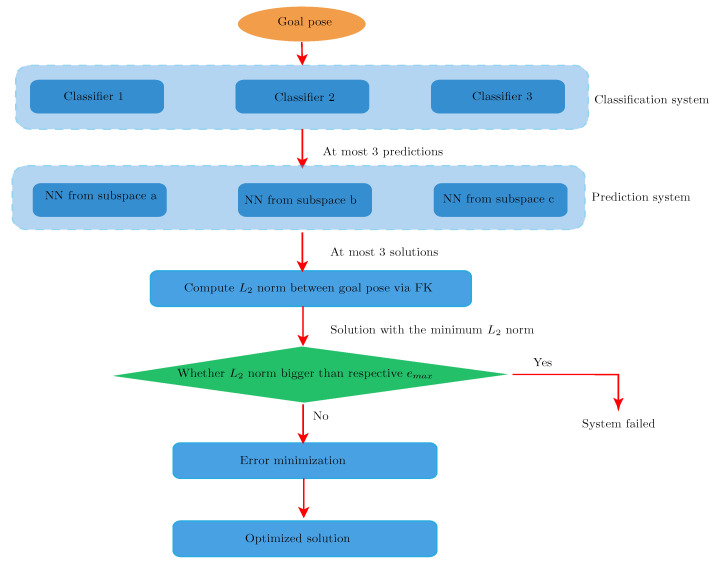
Workflow of the proposed IKP solution.

As illustrated in Figure 3, the main idea of the proposed algorithm is to approximate the non-linear function of IKP via MLP first, with a following error minimization process discussed in Section 3.2. Due to the complexity of our robot model, traditional NN models, such as those described in [16,25,35], behave unsatisfactorily. For the purpose of reaching an optimal performance with minimum training and computational cost, instead of resorting to advanced machine learning models, such as deep RNN or generative adversarial networks in [26], the workspace of Xarm6 is divided into smaller subspaces, the problem thus being handled piecewise. Details of implementation are introduced as follows.

### 4.1. Obtain the Training Data

During the implementation of neural networks, the first step is preparing the training data. Two possible ways can be adopted to generate the dataset mathematically: random generation and structured generation. For both methods, values of joint variables are required to be picked first in the joint space. Then, after being transformed into the workspace by means of Equations (2) and (3), a set of training data is prepared. The only difference between these two approaches is the way in choosing the value of each joint. In random generation, as the name suggested, the uniform distribution within the joint limitations is applied. By contrast, values of joint variables are progressively selected in structured generation, which is similar to the process of building a six layers “for” loop in C++.

Ideally, structured generation is a more reasonable way to describe the workspace systematically. However, a huge amount of training data always accompanies. For example, even by setting a relatively big step size of $10^{\circ }$, 34,012,224 sets of training data are obtained, which leads to extremely time-consuming learning. Additionally, within the framework proposed in [36], it is illustrated that with limited amount of data, random generation has a better performance for generalization of NN. Thus, in this study, the training sets are obtained with random generation, which is realized with the “random()” function in Matlab 2020b.

### 4.2. IKP Simplification and NN Training

As mentioned above, the performance of the trained neural network will be unimaginably poor if we fit the IKP of Xarm6 with a single NN directly. In this study, the strategies, joint space segmentation, are employed to simplify the problem and improve the performance.

On the one hand, although the IK function is extremely complicated in the whole joint space, if we only focus on a small subspace of IKP, we will obtain a relatively simple IK function and the NN would give reliable results. Based on this idea and experiments, we divide the joint space of Xarm6, as listed in Table 3. As a result, from the first joint to the last one, the allowed joint ranges of motion are separated into the following parts: 2, 2, 2, 3, 4, and 2, with 192 subspaces being generated in total.

It is noteworthy that, the amount of subspaces for joint space division is a hyper-parameter for the problem which requires tuning by experiments. An advice is applying an even division step, such as 60°, for each joint at first, randomly picking one divided subspace then, and fitting it with a simple MLP, such as our 3-layer MLP. This can help to figure out the complexity of IKP for a specific case, and also strategies for further adjustment.

Furthermore, after selecting the basic category of neural networks, there are also two kinds of configurations available for application, namely, single joint model and all joint model. The difference is vividly depicted by Figure 2, of which NNs can be regarded as all joint model for 5-DoF robots and single joint model for 6-DoF robots, respectively.

Since the single joint model approximates the solution with six sets of weights, the precision is expected to be improved, as proved in [16]. However, for six NNs in need, the overall training cost of the single joint model is several times higher than its all joint model’s counterpart. In a previous study, both NN models were investigated on Xarm6 for the optimal performance and the results coincide with aforementioned hypothesis. In addition, one interesting phenomenon was that the well-trained NN from all joint model can make reliable predictions for the first five joints. However, the result for the last joint was less accurate. Thus, instead of using the single or all joint model solely, as shown in Figure 2, a five output MLP was trained to predict the inverse kinematics solutions of the first five joints while the last joint was handled separately.

Additionally, the complexities of IK function also vary from different subspaces in Table 3. Hence, apart from the amount of neurons per layer being kept as a constant, the number of hidden layers and the training set is not fixed during the learning process. As a default setting, three hidden layers, 600 maximum epochs, and 80,000 sets of training data are configured during the learning process. If a subspace turned out to be challenging, the default configurations will be adjusted according to the performance with an upper bound as:

- The maximum iteration epochs: 2000;
- The maximum amount of hidden layers: 5;
- The maximum amount of training data: 200,000.

Other key parameters are given as:

- Amount of neurons per hidden layer: 20;
- Training method: L-M;
- Split of dataset: 70%, 15% and 15% for training set, cross validation set and test set, respectively.

### 4.3. Selection of the Optimum Prediction

Upon completing the above procedures, 192 sets of NNs representing different joint spaces were trained, and the same amount of predictions will be obtained given a goal pose. However, each potential solution can only be located in one subspace in Table 3, while only the prediction given by the corresponding neural network should be the desired solution. One straightforward means to extract it is computing the $L_{2}$ norm of position between the goal pose and the prediction. Apparently, the solution with the minimum norm is the most promising “correct one”, which is picked for further improvement. However, one fatal drawback of this method is the computational cost, attributing to the calling of all trained NNs for each prediction.

To tackle this, an intelligent classification system, composed of three deep NNs, are trained to determine which subspace the IK solution might locate given a desired pose. Architectures of these three networks are given in Table 4, where AF denotes the activation function, HL and OL being short for the hidden layer and output layer, respectively. Details of residual connection and batch normalization layer can be found in [37,38]. Here, different NN architectures are applied with the aim of insuring the performance to be more independent, that is, to avoid NNs making mistakes on the same sample.

With the integration of the classification system, the overall workflow is summarized in Figure 3. During applications, to achieve the highest reliability of the system, if NNs in the classification system give conflicting conclusions, instead of using the one with highest confidence, all unique predicted subspaces will be sent to the prediction system and the initial solution is selected by the $L_{2}$ norm. Considering the extreme cases—none output from the classification is right, the $L_{2}$ norm of the initial solution is further compared with $e_{max}$, defining $L_{2}$ as the maximum norm observed on test set during testing the respective MLP in the prediction system. A solution with a norm exceeding $e_{max}$ indicates the classification system may fail to function well and the “straightforward” mean should be considered.

## 5. Results and Discussion

### 5.1. Simplification vs. Non-Simplification

**Figure 4:**
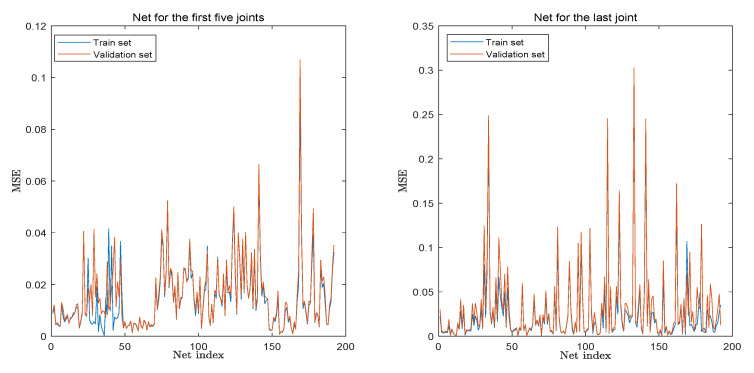
Final performance of the NNs for the prediction system.

**Figure 5:**
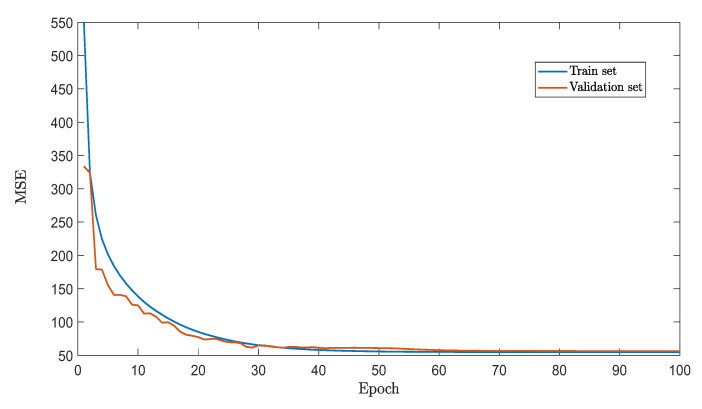
Training history for DNN.

The final performance of our trained net for the prediction system, indicated by MSE, is given in Figure 4. To illustrate why the simplification is indispensable, a deep neural network (DNN) is trained to solve the problem directly for comparison purposes. This DNN has 140 dense layers with batch normalization and residual connections. The total trainable parameters are 601,158, while the dataset for training and validation are 5,000,000 and 4800, respectively. The training history of the DNN is given in Figure 5.

From the figures, although our DNN is more complex than that in recent papers, such as [25,27], the model still ends with a MSE up to 50 after around 100 epochs. Further training is not considered since the model converges. By contrast, after simplification, the MSE for the prediction system is not more than 0.3, while the majority is below 0.1. The result clearly demonstrates the need for many networks in our prediction system.

### 5.2. Test with Random Pose

After training the proposed neural networks, we first tested our algorithm with random points in the workspace. In total, 25 sets of joint angles were generated randomly within each subspace, i.e., 4800 in total, with respective poses sought via Equation (2). These poses were applied as inputs of the algorithm in order to obtain the initial and optimized solutions. Performance of the classification system on training sets and test set is given in Table 5. In Table 6, five samples were picked from the test sets randomly as examples, with the corresponding solutions shown in bottom three rows.

**Figure 6:**
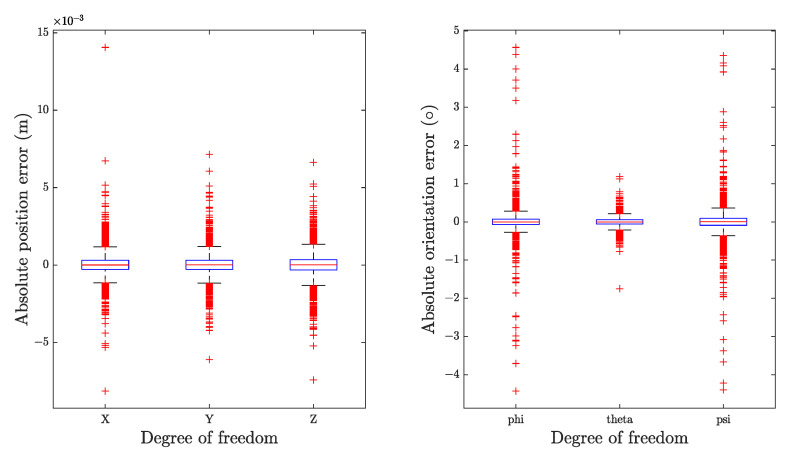
Absolute errors distribution of initial solutions in each direction.

**Figure 7:**
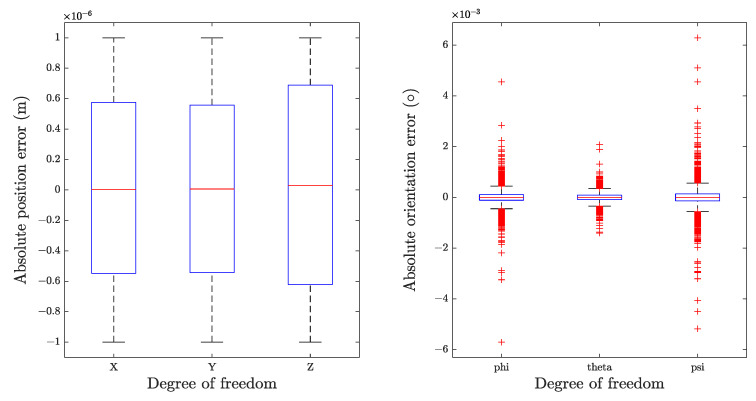
Absolute errors distribution of optimized solutions in each direction.

To evaluate the performance of the error minimization algorithm, both the initial and optimized solutions were transformed into the Cartesian Space. After subtracting the goal poses, the absolute errors of the initial and optimal solutions were obtained and plotted as boxplot in Figure 6 and Figure 7, respectively. Some error indicators were sorted and given in Table 7 and Table 8.

From the results, it is apparent that our trained neural networks manage to find an acceptable solution for IKP in all attempts. According to our statistics, without refining, 99.27% predictions are relatively accurate, reaching the precision of 5 mm for position and 2° for orientation. Error in this magnitude is sufficient for some collaborative robots, such as those for goods delivery. However, the percentage falls to 34.27% when the standard rises to 1 mm and 0.1°, and further reaches 1.96% at 0.1 mm and 0.1°. Apart from these, under certain circumstances, the error can reach up to 1.5 centimeters and 4.6°. This poses substantial challenges in tasks that require significantly high precision, such as surgery. Thus, further optimization is required. By contrast, with Newton–Raphson based error minimization procedure, all the final solutions meet our presupposed requirements. More than expected, even setting a small iterative criterion as 0.001 radians (0.0573°), all solutions have errors less than 0.01° for orientation.

One interesting thing is that, although the classification system fails to categorize all the test samples correctly, the system still manages to give reliable predictions all the time. These incorrectly labeled cases are further checked and details are sorted in Table 9. Comparing the initial solution norm with respective $e_{max}$, it is surprised to find the influence of utilizing MLP from mismatched subspace was negligible since the former is much more smaller. An explanation for this is the IK function under some subspaces are extremely similar and the classifiers are thus prone to make mistakes in these cases. However, similar IK functions also result in analogical weights of the prediction system for these subspaces, making the impact insignificant as a result. The existence of this phenomenon definitely makes our algorithm more efficient than it appears to be.

### 5.3. Trajectory Tracking

Apart from random data, to simulate the practical motion of the robot in Cartesian space, the algorithm is verified with structural data, by tracking a curve in the three-dimensional workspace. The goal trajectory is a fraction of a helical path, which is frequently met in industrial fields [25]. While the goal positions [x,y,z] are specified by the parametric equations as

(13) $$\begin{array}{cc} \; & x=a\cos t\\ \; & y=b\sin t\\ \; & z=ct \end{array}$$

where *a*, *b*, and *c* are constants, *t* being the variable for time. The desired orientation is selected and kept fixed along the whole trajectory to guarantee the existence of solutions within the joint limitations.

**Figure 8:**
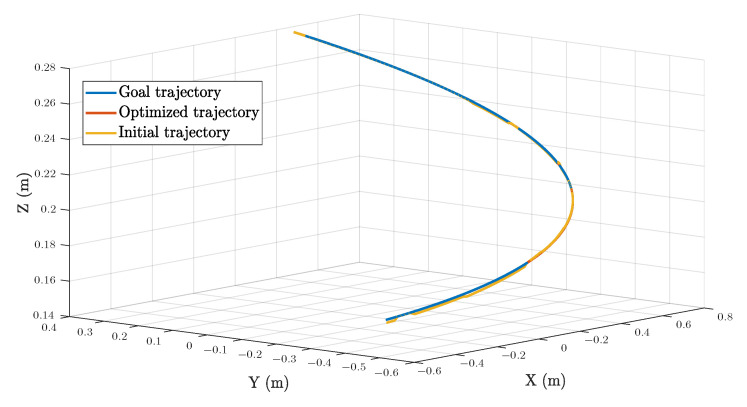
Trajectory tracking of a 3D curved line.

**Figure 9:**
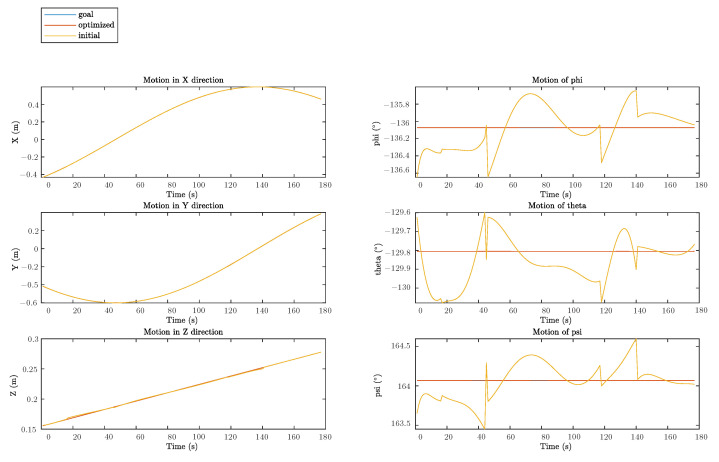
Decoupled motions in each direction.

**Figure 10:**
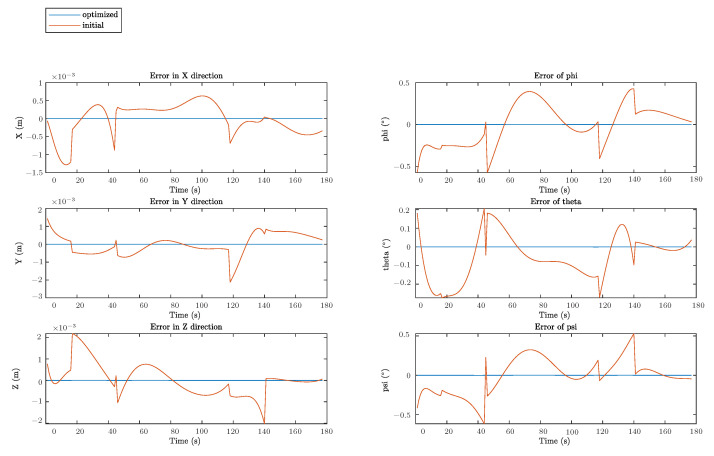
Errors for translation and rotation in each direction.

Plot of the goal trajectory, as well as that originating from the initial and optimized solutions, is shown in Figure 8. Respective decoupled motions, including rotation and translation in each direction, are illustrated in Figure 9, whereas Figure 10 describes the error of pose w.r.t. time.

As can be seen from Figure 8 and Figure 9, there are some observable deviations between trajectories from the initial solutions and the goal poses during the whole process. Discrepancies at the beginning, i.e., before the first 40 seconds, are the most distinct where the maximum error can be up to 2 mm. These distinctions in positions are mainly caused by the noise on the Z direction. For rotations, from Figure 9 and Figure 10, the errors seem to be more irregular, oscillating between $-0.4^{{}^{\circ}}$ and $0.4^{{}^{\circ}}$ all the time. Meanwhile, the impact of the proposed error minimization algorithm is observed more clearly from these results. After improvement in any plots of trajectories, discrepancies between the goal trajectory and its optimized counterpart are negligible, while respective errors almost coincided with the straight line y=0 in the error plot.

**Figure 11:**
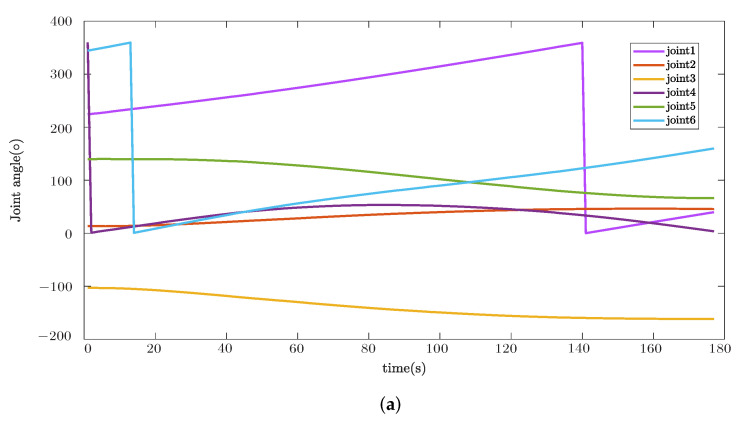

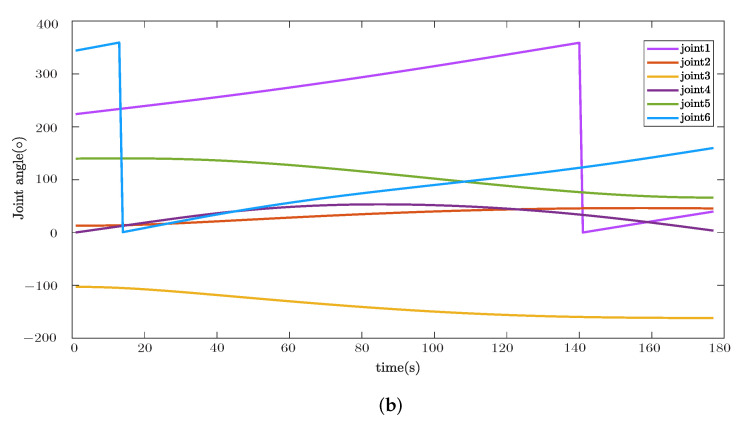
Joint motion results for initial and optimized solution during trajectory tracking task. (**a**) Joint motion of initial solution; (**b**) Joint motion of optimized solution.

Additionally, joint motion results during the trajectory tracking task are plotted in Figure 11. Since the discontinuities in figures are caused by passing from $360^{{}^{\circ}}$ to $0^{{}^{\circ}}$, which is a continuous motion in reality, it is proved that the system can give smooth solutions for a specific trajectory.

### 5.4. Efficiency and Singularity

In addition to accuracy, the efficiency of the inverse kinematics algorithm, indicated by the computation time, was also an important factor in practical applications. A comparison of the proposed algorithm with the research works in [21,25] is performed, on position and orientation errors, as well as processing time on initial and optimized solution. Comparison results are presented in Table 10, where NR denotes the error minimization method resorted to Newton–Raphson in this paper, GA represents the genetic algorithm in [21], and EF is the optimization process based on error feedback in [25].

Compared with the five-DoF and six-DoF decoupled robots in [19,39], respectively, the six-joint robot with offset wrist being studied in this work brings in more challenges for solving the IKP. Nevertheless, from Table 10, it can be seen that our algorithm is capable of achieving a similar or better precision with the highest efficiency. Moreover, although the computational time on the optimized process is not given for GA, considering the extremely low computational cost for NNs to give predictions, the processing time can be inferred to be more than 0.2 s. Noticing the 0.2275 s optimization time for MEF, apparently, NR is the optimal one to serve as a real-time error minimization method.

It is noteworthy that though the solution of IKP can be directly found via the NR method with a random initial guess, two drawbacks arise. First, a bad random initialization may lead to a solution outside the joint limitation. Second, since the algorithm is based on the inverse of the Jacobian matrix, it might suffer from the singularity problems. In terms of utilizing Equation (12) as an error minimization approach, these defects can be, to a large extent, avoided. This is partially due to that the prediction system behaves just like an intelligent initial guess supplier and can always provide a suitable start point for the iterative process. To be more specific, for one thing, since NNs learn from practical data, the prediction of a well trained system, the initial solution, will be close to an executable solution. As a result, the optimal solution will likely to fall into the local minimum around it and the final solution is thus feasible. For another, the Jacobian matrix cannot suddenly be illness. If the initial solution is relatively accurate, only a small adjustment will be required for each joint to reach its practical counterpart during optimization and the iterative process is, hence, unlikely to pass across any illness region. The only exception is the goal pose being at or infinitely close to a singular configuration, which should be avoided during practical applications.

To verify the above hypothesis, the NR algorithm was tested to solve the same IKPs in Section 5.2 with random initial guesses. During the searching process, the following cases might happen:

1. Failures due to the singularity problem.
2. Failures due to joint limitation. The solution exceeds joint limitation.
3. Success. The algorithm gives a desired answer.

While in case 3, the algorithm directly stops, for case 1 and 2, the algorithm will restart at another start point until the target is completed. The test was repeated three times. Frequencies of different types failures designating with $F_{singular}$ and $F_{limit}$, as well as other useful results, are sorted and given in Table 11.

Although the amount of target poses is 4800 overall, from the contents in Table 11, the algorithm takes around 5 times more steps to find the respective solutions due to its drawbacks. Meanwhile, from the results in Section 5.2, with the initial guesses from NNs, both failures never occur during the optimization procedure. Thus, it can be concluded the initial guess from NN does make the NR algorithm more robust during the searching process. What is more, as the algorithm only needs to be run once for each target, the overall efficiency is also boosted around 4 times.

## 6. Conclusions

Inverse kinematics is a fundamental problem in robotics. Since the degree of non-linearity of the kinematic equations increases sharply with the number of DoFs, seeking inverse kinematics solutions for general robots with high orders is extremely challenging. In this paper, a novel NN-based approach composed of three key parts—the prediction system, the classification system, and the opitmization system—is presented to solve the IKP for the general six-axis robot. Within our framework, for the prediction system, the joint space is first divided into some smaller subspaces and a sets of MLP are applied to fit the inverse kinematics function in pieces. For each pair of neural networks, a five-output MLP is used to predict values of the first five joints, while another single output MLP is trained to approximate the last joint separately. Subsequently, to reduce the computational cost of making predictions, a classification system is trained to determine which foregoing MLP, representing difference joint spaces, should be selected given a desired pose. Considering some tasks requiring significantly high precision, such as micrometer level, the initial solution from the prediction system is further refined by a numerical iterative process originating from Newton–Raphson method. The feasibility of our algorithm is first tested by 4800 random desired poses with a following task of drawing a curve in the workspace to simulate the practical applications. Comparison are made with other approaches in literature to reveal the advantages of our algorithm.

The results from the random pose and trajectory tracking test show that with our workflow, the system can make reliable and consecutive predictions with the preset goal—a position and orientation error less than 0.001 mm and 0.001 rad respectively. Meanwhile, thanks to the classification components, the processing speed of our approach, 0.0218 s per target on average, is satisfying. Furthermore, the proposed NR-based error minimization method is verified to be extremely robust towards the singularity problem with an efficiency being dozens of times that of the heuristic methods reported in literature.

There is some room for the methodology improvement and simplification of the training process is the preoccupation. At this moment, the training process of our methodology is a little tedious since the step size for the workspace division is selected by experiments. We will focus on a systematic approach to select this hyper-parameter efficiently in the future. The future work also includes experimental test for the methodology validation.

## Figures and Tables

**Table 1:** Comparison of the previous and present work.

	Reference	Robot Model	Analytical Solution	Accuracy
ANN	[16,20,22,23,24,25,27,28,30]	Low-DoF or 6-DoF decoupled	Yes	low to extremely high
RNN	[18,21]	6-DoF decoupled	Yes	fair to extremely high
RFB	[17,19]	6-DoF decoupled	Yes	fair to high
GAN	[26]	Low-DoF	Yes	high
Our work		6-DoF general robot	No	extremely high

**Table 2:** D-H parameters of Xarm6.

Joint Number	*a* (mm)	α (°)	*d* (mm)	θ
1	0	−90	267	$\theta_{1}$
2	289.5	0	0	$\theta_{2}$
3	77.5	−90	0	$\theta_{3}$
4	0	90	343.5	$\theta_{4}$
5	76	−90	0	$\theta_{5}$
6	0	0	97	$\theta_{6}$

**Table 3:** Joint space division of Xarm6.

Joint Number	Goal Range	After Dividing
1	(0, 360°)	(0, 180°) (180, 360°)
2	(0, 90°)	(0, 60°) (60, 90°)
3	(−180, −90°)	(−180, −120°) (−120, −90°)
4	(0, 180°)	(0, 60°) (60, 120°) (120, 180°)
5	(0, 180°)	(0, 45°) (45, 90°) (90, 120°) (120, 180°)
6	(0, 360°)	(0, 180°) (180, 360°)

**Table 4:** Architecture of NNs for classification system.

No.	1	2	3
HL No.	6	20	30
Hidden unit	35	35	35
AF in HL	Relu	Relu	Relu
AF in OL	softmax	softmax	softmax
Trainset	576,000	960,000	768,000
Optimizor	Adams	Adams	Adams
Features	Simple MLP	MLP with residual block	MLP with residual block and batch normalization

**Table 5:** Performance of the classification system.

	NN 1	NN 2	NN 3	Overall Classification System
Train set	94.50%	97.87%	98.53%	N/A
Test set	93.60%	95.40%	96.25%	99.50%

**Table 6:** Example of random data test.

	No	*x* (m)	*y* (m)	*z* (m)	ϕ (rad)	θ (rad)	ψ (rad)
Goal Pose	1	−0.64142	0.22652	0.22762	−0.10588	−2.08249	2.34062
	2	0.45064	0.15015	0.63200	−2.54416	−0.76447	−2.30342
	3	0.19978	0.55744	0.51288	−1.06619	−1.83045	2.78188
	4	0.33833	0.42766	0.28287	0.40171	−3.01724	−0.99056
	5	−0.24216	0.39147	−0.17398	1.78176	−2.12424	2.17019
	**No**	** $q_{1}$ **	** $q_{2}$ **	** $q_{3}$ **	** $q_{4}$ **	** $q_{5}$ **	** $q_{6}$ **
Exact Solution	1	160.46907	37.91946	−135.87082	18.62891	39.46047	112.83264
	2	24.29447	11.89334	−153.69625	53.15470	8.58257	182.38461
	3	64.38381	14.31783	−145.74087	54.39600	75.48167	120.27973
	4	50.64234	30.28887	−156.69763	4.48408	132.64058	153.73039
	5	117.71272	57.93731	−121.66049	15.36286	120.12755	320.55779
Initial Solution	1	160.44294	37.90550	−135.93878	18.59329	39.40203	112.92553
	2	24.26964	11.93607	−153.77572	53.15427	8.66457	182.47650
	3	64.36072	14.37610	−145.80470	54.32014	75.51120	120.29442
	4	50.57437	30.29154	−156.75669	4.56464	132.62676	153.75462
	5	117.75096	58.00550	−121.67093	15.17564	120.30039	320.64783
Optimized Solution	1	160.46905	37.91945	−135.87089	18.62888	39.46041	112.83273
	2	24.29433	11.89359	−153.69672	53.15470	8.58306	182.38515
	3	64.38376	14.31797	−145.74103	54.39582	75.48174	120.27977
	4	50.64217	30.28888	−156.69777	4.48428	132.64055	153.73045
	5	117.71276	57.93739	−121.66051	15.36263	120.12776	320.55790

**Table 7:** Error indicators for initial solution.

	$e_{x}$ **(m)**	$e_{y}$ **(m)**	$e_{z}$ **(m)**
mean absolute error	0.0004	0.0005	0.0005
minimum absolute error	6 × $10^{-8}$	3.6 × $10^{-7}$	1.9 × $10^{-7}$
maximum absolute error	0.014059	0.007147	0.007430
	**$e_{\phi }$ (°)**	**$e_{\theta }$ (°)**	**$e_{\psi }$ (°)**
mean absolute error	0.1344	0.0812	0.1687
minimum absolute error	6.241 × $10^{-5}$	1.8 × $10^{-5}$	3.946 × $10^{-5}$
maximum absolute error	4.5682	2.0621	4.3986

**Table 8:** Error indicators for optimized solutions.

	$e_{x}$ **(m)**	$e_{y}$ **(m)**	$e_{z}$ **(m)**
mean absolute error	5.461$\times 10^{-7}$	5.462$\times 10^{-7}$	5.884$\times 10^{-7}$
min absolute error	1.612$\times 10^{-10}$	5.875$\times 10^{-11}$	3.483$\times 10^{-10}$
max absolute error	9.998$\times 10^{-7}$	9.994$\times 10^{-7}$	9.998$\times 10^{-7}$
	**$e_{\phi }$ (°)**	**$e_{\theta }$ (°)**	**$e_{\psi }$ (°)**
mean absolute error	1.868$\times 10^{-4}$	1.233$\times 10^{-4}$	2.572$\times 10^{-4}$
min absolute error	1.209$\times 10^{-7}$	2.025$\times 10^{-8}$	1.059$\times 10^{-8}$
max absolute error	5.7$\times 10^{-3}$	2.1$\times 10^{-3}$	6.3$\times 10^{-3}$

**Table 9:** Mislabeled cases of the prediction system.

Sample No.	True Label	Classification System Output	Initial Solution Error ($L_{2}$ Norm)	Criterion ($e_{\max}$)
73	3	11	9.6 × $10^{-7}$	1.67 × $10^{-4}$
345	14	22	1.005 × $10^{-5}$	1.36 × $10^{-3}$
372	15	111	1.318 × $10^{-5}$	6.87 × $10^{-5}$
381	16	8 and 15	3.3 × $10^{-7}$	1.26 × $10^{-4}$
610	25	73 and 121	4.07 × $10^{-6}$	1.18 × $10^{-3}$
1723	69	70	2.5 × $10^{-7}$	8.15 × $10^{-5}$
1836	74	82	2.58 × $10^{-6}$	8.84 × $10^{-4}$
1849	74	76	4.92 × $10^{-6}$	9.45 × $10^{-4}$
2112	85	87	3.7 × $10^{-7}$	3.65 × $10^{-4}$
2200	88	86	2.153 × $10^{-5}$	6.03 × $10^{-4}$
2480	100	148	2.07 × $10^{-6}$	2.99 × $10^{-4}$
2649	106	105	1 × $10^{-8}$	5.44 × $10^{-4}$
3019	121	122	1.3 × $10^{-6}$	9.81 × $10^{-4}$
3156	127	31	7 × $10^{-8}$	4.65 × $10^{-4}$
3233	130	178	1.347 × $10^{-5}$	1.43 × $10^{-3}$
3235	130	122	1.3 × $10^{-5}$	9.81 × $10^{-4}$
3246	130	122	4.801 × $10^{-5}$	9.81 × $10^{-4}$
3333	134	182 and 190	4.4 × $10^{-7}$	1.28 × $10^{-4}$
3352	135	143	1.007 × $10^{-5}$	7.36 × $10^{-3}$
3478	140	139	1.58 × $10^{-6}$	5.41 × $10^{-4}$
3514	141	189	4 × $10^{-7}$	1.44 × $10^{-3}$
3536	142	190	5.13 × $10^{-6}$	6.36 × $10^{-4}$
4052	163	67	2.6 × $10^{-7}$	1.30 × $10^{-4}$
4403	177	179	6.4 × $10^{-7}$	9.00 × $10^{-5}$

**Table 10:** Comparison of three error minimization approaches.

	Error for Position	Error for Orientation	Time on Initial Solution	Time on Optimization	Total
NR	0.001 mm (max)	0.01° (max)	0.0159 s	0.0059 s	0.0218 s
GA	0.0037 mm (max)	N/A	N/A	N/A	0.28 s
EF	0.0054 mm (mean)	1.1154° (mean)	0.0075 s	0.2775 s	0.285 s

**Table 11:** Results of NR with random goal poses tests.

	$F_{\mathrm{singular}}$	$F_{\mathrm{limit}}$	Total Steps	Run Time (s)	Time/Solution (s)
Test 01	53	18927	23780	481.52	0.1003
Test 02	74	18293	23167	475.345	0.099
Test 03	79	18423	23302	476.243	0.0992

## Data Availability

Not applicable.
